# Prolonged survival after sequential multimodal treatment in metastatic renal cell carcinoma: two case reports and a review of the literature

**DOI:** 10.1186/1752-1947-6-303

**Published:** 2012-09-14

**Authors:** John Syrios, Georgios Kechagias, Nicolas Tsavaris

**Affiliations:** 1 Department of Pathophysiology, Oncology Unit, Laikon General Hospital, Athens University School of Medicine, 75 Mikras Asias street, Athens, 11527, Greece

**Keywords:** Metastatic renal cell carcinoma, prolonged survival, sequential therapy, quality of life

## Abstract

**Introduction:**

In this case series and short review of the literature, we underline the impact of nephrectomy combined with sequential therapy based on cytokines, antiangiogenic factors, and mammalian target of rapamycin inhibitors along with metastasectomy on overall survival and quality of life in patients with metastatic clear cell renal carcinoma.

**Case presentation:**

In the first of two cases reported here, a 53-year-old Caucasian man underwent a radical left nephrectomy for renal cell cancer and relapsed with a bone metastasis in his right humerus. He was treated with closed nailing and cytokine-based chemotherapy. For 5 years, the disease was stable and he had great improvement in quality of life. Subsequently, the disease relapsed in his lymph nodes, lung, and thorax soft tissue. He was then treated with antiangiogenic factors and mammalian target of rapamycin inhibitors. The disease progressed until September 2009, when he died of allergic shock during a blood transfusion, 9 years after the initial diagnosis of renal cell cancer.

In the second case, a 54-year-old Caucasian man underwent a radical left nephrectomy for renal cell cancer. A year later, the disease progressed to his neck lymph nodes, and cytokine-based chemotherapy was initiated. While he was on cytokines, a solitary pulmonary nodule appeared and he underwent a metastasectomy. Nine months later, magnetic resonance imaging of his brain revealed a focal right occipitoparietal lesion, which was resected. After two years of active surveillance, the disease relapsed as a pulmonary metastasis and he was treated with an antiangiogenic factor. Further progressions presenting as enlarged axillary lymph nodes, chest soft tissue lesions, and thoracic spine bone metastases were sequentially observed. He then received a first-generation mammalian target of rapamycin inhibitor, an antiangiogenic factor, and later a second-generation mammalian target of rapamycin inhibitor and palliative radiotherapy. Ten years after the initial diagnosis of renal cell cancer, his disease is stable and he is on a third antiangiogenic factor and leads an active life.

**Conclusions:**

One multidisciplinary approach to patients with metastatic renal cell cancer combines nephrectomy, metastasectomy, and radiotherapy (when feasible) with medical therapy based on cytokines and targeted treatment employing agents inhibiting angiogenesis, other receptor tyrosine kinases, and mammalian target of rapamycin. This approach could prolong survival and improve quality of life.

## Introduction

Renal cell carcinoma (RCC), a relatively common malignancy, accounts for 2% to 3% of all malignant tumors in adults [1]. In Europe, it has a rising incidence and represents the third most prevalent urologic malignancy: RCC is diagnosed in 40,000 patients each year. Patients with untreated metastatic RCC (mRCC) have a 5-year survival rate of only 0% to 18%, whereas patients with RCC of any stage have a 5-year survival rate of 62% [2,3], indicating an aggressive malignancy. At the time of diagnosis, one third of patients present with locally advanced or metastatic disease and one third of patients undergoing cytoreductive nephrectomy will experience relapse and develop metastasis [4]. In these settings, first-line medical treatment is recommended. The current management of mRCC is challenging given the various therapeutic options available after the development of several new targeted drugs. Until relatively recently, cytokine treatment with interferon-alpha (IFN-α) and interleukin-2 was the gold standard of treatment. Only after the approval of antiangiogenic agents that directly inhibit vascular endothelial growth factor (VEGF) (bevacizumab), others that target VEGF receptors and tyrosine kinase receptors (sorafenib, sunitinib, pazopanib, and axitinib), and factors that inhibit the mammalian target of rapamycin (mTOR) (temsirolimus and everolimus) did patients with mRCC experience higher response rates and prolonged survival [5]. With the development of these agents, the progression-free survival (PFS) has practically doubled, and up to 30% of patients achieve partial remission [2]. According to emerging evidence, administering these drugs sequentially provides a further prolongation of PFS and a clear clinical benefit [6].

## Case presentation

### Case 1

**Figure 1:**
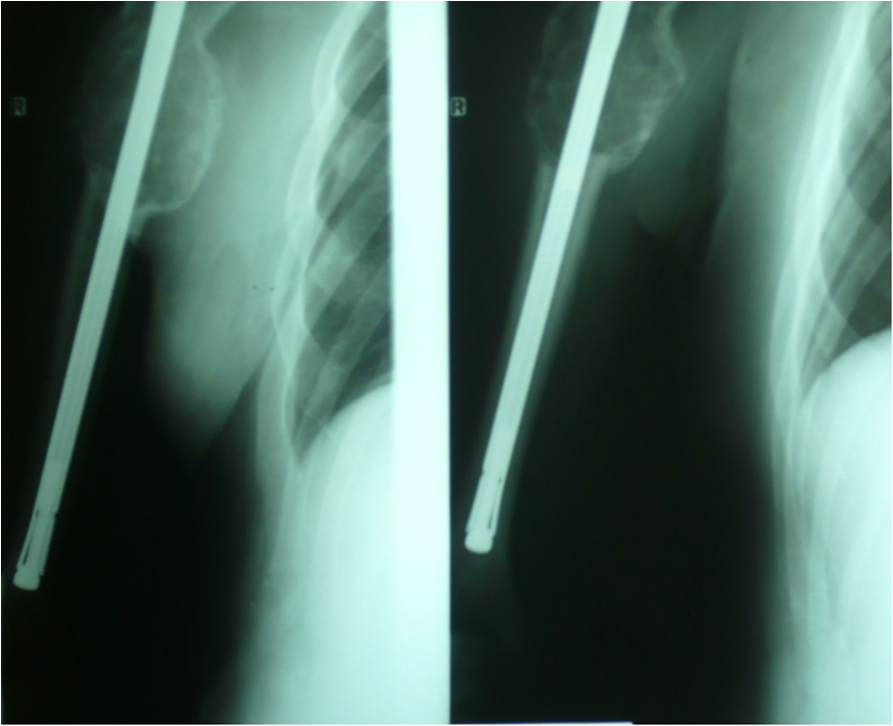
Closed nailing of the right humerus.

**Figure 2:**
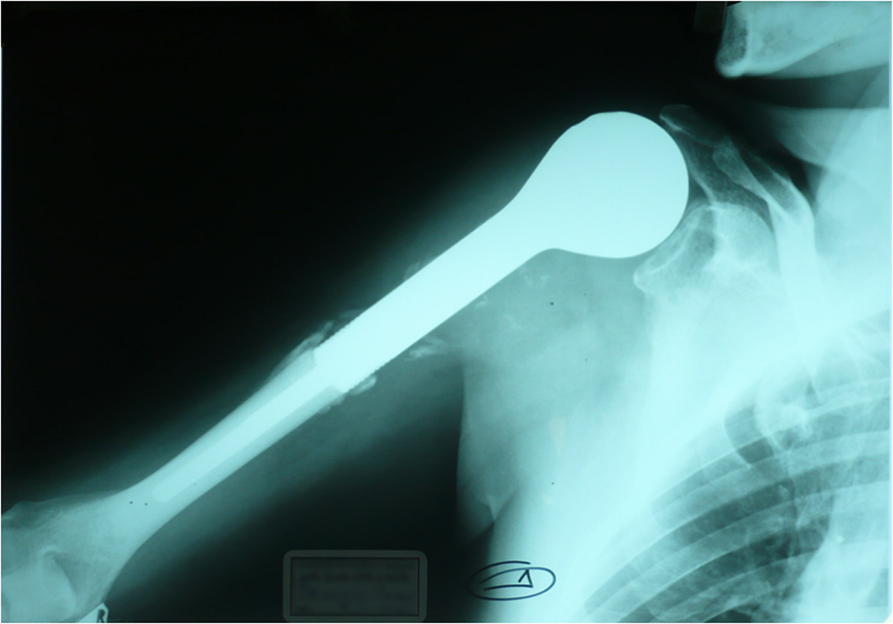
Right humerus nail replacement.

In September 2000, a 53-year-old Caucasian man who was a heavy smoker underwent a radical left nephrectomy for a grade 1, stage I, renal clear cell carcinoma revealed on a routine abdominal ultrasound exam. In July 2001, he presented with a dull pain in his right humerus which rapidly worsened. The pain caused the patient, a professional musician, such discomfort that he was forced to stop playing his instrument (the bouzouki, a stringed instrument from Greece), and strong opioids were required for pain control. The results of a computed tomography (CT) scan of his right humerus and a technetium bone scan showed a solitary bone metastasis. He was treated with closed nailing of his right humerus, but total resection of the metastasis was not achieved (Figure 1). He was put on cytokine-based chemotherapy from May 2002 to February 2003. The chemotherapy consisted of IFN-α2α 6MU administered subcutaneously three times per week, recombinant human interleukin-2 at a dose of 9×10^6^IU subcutaneously for 4 weeks followed by 1 week of rest, and vinorelbine 30mg/m^2^ and zolendronic acid 4mg every 21 days. Then he underwent a right humerus nail replacement with 10-fraction radiotherapy in order to render his extremity pain-free and capable of weight-bearing (Figure 2). He was offered physiotherapy but declined. He received IFN-α treatment for a further 4 months and, notably, resumed playing the bouzouki, which requires significant upper-extremity dexterity, attesting to a dramatic improvement of his symptoms. His disease was stable and he led an active life from September 2003 to June 2008, when a chest CT scan revealed several enlarged subcarinal, left hilar, and axillary lymph nodes. He was treated with sunitinib at 50mg/day for 4 weeks with a 2-week washout phase along with vinorelbine 30mg/m^2^, bevacizumab 200mg, and zolendronic acid every 21 days. A partial response was observed until February 2009, when a chest CT scan revealed several pulmonary nodes consistent with metastases. Therapy was switched to temsirolimus at 25mg weekly until June 2009, when he experienced further deterioration with pleural effusions and a soft tissue metastasis of his thorax. He received sorafenib at 800mg/day along with bevacizumab at 200mg weekly and developed a grade III anemia that impacted negatively on his performance status and that was treated with erythropoiesis-stimulating agents and blood transfusions. The disease progressed until September 2009, when he died of allergic shock during a blood transfusion, 9 years after the initial diagnosis of RCC.

### Case 2

A 54-year-old Caucasian man, a civil engineer, presented with acute urinary retention in July 2002. An abdominal CT scan showed a mass in his left kidney, and he underwent an uneventful radical left nephrectomy for a pT2, grade IV renal clear cell carcinoma. On August 2003, a physical examination revealed an enlarged supraclavicular lymph node, which was histologically proven to be RCC. He was put on cytokine-based chemotherapy – IFN-α 6MU subcutaneously 3 days per week, and docetaxel 60mg and vinorelbine 50mg every 21 days – until November 2004, when a nodule at the upper lobe of his right lung was found on a chest CT scan. The biopsy of the solitary pulmonary nodule confirmed an RCC metastasis, and he underwent a metastasectomy. On September 2005, a magnetic resonance tomography scan of his brain revealed a focal lesion, 3cm in diameter, at the right occipitoparietal region. Two months later, the solitary brain metastasis was resected and was histologically proven to be RCC. In March 2007, a new solitary pulmonary nodule in his lower right lobe was found, and sunitinib at 50mg/day for 4 weeks with a 2-week washout phase was administered. The disease was stable until February 2008, when a chest CT scan revealed bilateral enlargement of axillary lymph nodes, a lesion with soft tissue density in the anterior aspect of the right pulmonary artery, and another one in the hilus of the left lung. Owing to his progressive disease, temsirolimus at 25mg weekly was initiated. Three months after temsirolimus initiation, intravenous zolendronic acid every 21 days was added because of the appearance of new bone metastases involving thoracic vertebrae. A partial response was observed until October 2008, when the soft tissue density lesions progressed and treatment with sorafenib 400mg/day was initiated. The disease was stable until June 2010, when a new pulmonary nodule appeared and the bone metastases became painful. Then the patient was treated with a second-generation mTOR inhibitor, everolimus 10mg by mouth every day, and 6-fraction palliative radiotherapy was applied to the painful spine bone metastases at a total dose of 24Gy. His disease was rendered stable for a year, when a new pulmonary metastasis appeared. The patient has since been receiving pazopanib at 800mg/day, resulting in stable disease to the present day. Ten years after the diagnosis of RCC, he leads an active life and only moderate bone pain prevents him from doing strenuous activity.

## Discussion

Clear cell RCC accounts for 70% to 75% of all histologic subtypes of RCC [7]. It may progress insidiously over a span of years, but once metastasis becomes evident, the 5-year survival rate declines sharply from more than 50% to 6% [8]. Nephrectomy or nephron-sparing surgery has been proven to be of benefit and is usually performed even in the setting of mRCC, except for poor-prognosis patients, according to criteria of the Memorial Sloan-Kettering Cancer Center [9]. Both cases presented here underwent a nephrectomy with curative intention since their disease was not metastatic at diagnosis, and both were meticulously followed up with given that there was no indication for any adjuvant treatment. Once metastases are present, lungs are commonly affected by a single metastasis (30.4%) or multiple metastases (75.6%), whereas bones are affected in 14% of patients with mRCC. Solitary bone metastasis, mostly a lytic lesion, may present in up to 26% of mRCC cases and confers a 5-year survival rate of 11% [10]. The most common locations of bone metastases from RCC are the spine, pelvis, femur, scapula, and humerus. Since they are highly destructive vascular lesions, they pose significant surgical challenges due to the risk of life-threatening hemorrhage and are resistant to other treatments. However, patients with a solitary bone metastasis have the most favorable overall survival [11]. Althausen *et al*. [12] report that those patients with solitary osseous metastasis and the longest interval between the diagnosis of RCC and the diagnosis of the metastasis have a relatively favorable prognosis and these carcinomas should be treated as radically as possible, whereas Kavolius *et al*. [13] report that resection of solitary metachronous RCC metastases from RCC is associated with a 5-year survival rate of 35% to 50%. Case 1 had a metachronous right humerus solitary metastasis, which appeared 1 year after the diagnosis of RCC. Given the relatively favorable clinical setting and the fact that the patient experienced a very significant deterioration in quality of life and even had to stop working, he underwent an orthopedic surgical procedure followed by radiotherapy. Indeed, implant stability and local control of disease were achieved and his extremity was rendered pain-free and capable of weight-bearing. Subsequently, he received cytokine-based chemotherapy – which consisted of IFN-α 6MU subcutaneously, interleukin-2 at a dose of 9×10^6^IU, vinorelbine 30mg, and zolendronic acid 4mg every 21 days – because total resection of the metastasis was not feasible. The outcome was impressive since the patient, a musician, resumed his job, which required rapid, fine, and coordinated upper-extremity movements.

Lung metastases are also a relevant therapeutic challenge. The 5-year survival rate after complete resection of pulmonary metastasis from RCC is up to 60% [3]. Volkmer *et al*. [14] report that the survival rate is significantly higher after resection of pulmonary metastases than after resection of extrapulmonary metastases. In case 2 presented here, a solitary metastatic nodule at the upper lobe of his right lung appeared 20 months after the initial diagnosis of RCC. The presence of a solitary metastasis, a long interval between the diagnosis of RCC and the diagnosis of metastasis, and a good performance status indicated a favorable clinical setting for resection, and the patient underwent a metastasectomy.

Brain metastases usually develop as a late manifestation of RCC and pose an increasing challenge to oncologists. Pomer *et al*. [15] defined a subgroup of patients who had a significant benefit from aggressive treatment of brain metastases. Patients with a metachronous appearance of brain metastases more than 1 year after nephrectomy, good performance status, age of less than 50 years, minimal or no neurological deficit, and minimal extracranial metastases showed a trend toward improved survival with metastasectomy. Also, the authors showed that surgical treatment of recurrent brain tumors yielded an additional median survival advantage of 8 months as compared with untreated patients [15]. Case 2 had a focal lesion at the right occipitoparietal region a year after metastasectomy for the lung lesion was performed. He had favorable clinical attributes as defined in the study by Pomer *et al*. Therefore, the solitary brain metastasis was resected.

We consider that the long survival of the two cases could be attributable to the positive patient profile that favored metastasectomies and to the sequential medical therapy, which consisted of cytokines, tyrosine kinase receptor inhibitors, and inhibitors of the mTOR.

Cytokines were considered the cornerstone of the treatment of RCC, and IFN-α and interleukin-2 yielded durable, albeit rare, complete remissions in certain subgroups [16]. Systemic therapies employed in patients with mRCC included classic cytotoxic agents such as vinorelbine and gemcitabine along with cytokines with some promising results [17,18]. After the favorable setting of the disease was taken into consideration, both cases of the study were offered first-line cytokine-based therapy along with standard chemotherapy agents (the clinical practice at the time) in order to achieve a prolonged overall survival.

According to clinical evidence, sequential targeted therapy is recommended in patients with mRCC and confers improved responses and prolonged survival. Bellmunt *et al*. [5] report the goals of the sequential therapy: achieving a treatment continuum (with the intention of maintaining patients on treatment without progression for as long as possible), attaining full dose intensity of targeted agents, ensuring that patients are exposed to optimal drug levels, minimizing adverse effects, and maximizing clinical benefit. Additionally, targeting different pathways through sequential therapy may offer an advantage in terms of overcoming resistance to individual agents [19].

Response rates to systemic therapy with cytokines vary from 5% to 20% and there are significant adverse effects [20]. Thus, several targeted agents that have a safer toxicity profile have since been developed. Bevacizumab, a VEGF monoclonal antibody that inhibits angiogenesis, may be used along with IFN-α as a first-line treatment in patients with favorable or intermediate disease profile risk, offering a prolonged PFS [21]. Another family of approved agents that target angiogenesis and other molecular pathways include the multikinase inhibitors. Sunitinib inhibits the receptor tyrosine kinases (RTKs) VEGF, VEGFR2, platelet-derived growth factor receptor (PDGFR), FLT-3, and c-KIT and prolongs the PFS in the first-line setting [22]. Sorafenib inhibits multiple RTKs, including VEGF-2, FLT-3, PDGF, FGFR-1, and Raf; has yielded a PFS advantage in the second-line setting; and is recommended after cytokine failure [23]. Pazopanib inhibits VEGFR, PDGFR, and c-Kit and may be used either as a first-line regimen or after cytokine failure [24]. In patients with disease progression on first-line therapy, axitinib, a VEGFR inhibitor, is also recommended.

Another pathway involved in RCC growth, proliferation, angiogenesis, and potential for metastasis is the mTOR. Temsirolimus inhibits mTOR in the PI3K-Akt pathway and is a recommended first-line regimen for the poor-risk patient group [25]. In anti-VEGF refractory mRCC, everolimus, a novel orally administered mTOR inhibitor, is indicated [26]. Many targeted drugs have been developed over the last decade, and others, including etaracizumab, vorinostat, XL880, and infliximab, are currently under study.

In terms of toxicity, patients treated with tyrosine kinase inhibitors may experience several adverse effects such as fatigue, hypertension, proteinuria, cardiac toxicity, hypothyroidism, hematological effects, hand-foot syndrome, mucositis, and gastrointestinal toxicities. The VEGF antibody-cytokine combination presents a different pattern of toxicity, including gastrointestinal perforation, bleeding, thromboembolic events, proteinuria, anorexia, and fever. The adverse event profile of mTOR includes hyperglycemia, hyperlipidemia, asthenia, hematological toxicity, pneumonitis, infections, and mucositis [27].

Case 1 experienced only mild hypertension attributed to sunitinib that was successfully treated with an angiotensin II receptor blocker. However, he experienced grade III anemia when therapy was switched to temsirolimus and was treated with erythropoiesis-stimulating agents and blood transfusions. The cause of death of this patient, an uncontrollable allergic reaction to a blood transfusion, could be considered an indirect effect of temsirolimus-induced anemia.

Case 2 developed clinical hypothyroidism 6 months after sunitinib initiation and was offered levothyroxine. Temsirolimus was well tolerated, and sorafenib caused only mild asthenia, grade I myelosupression, and hyperlipidemia. Treatment with pazopanib caused anorexia and grade II diarrhea. Notably, in both cases, no treatment delay or dose reduction was needed.

The combination of these agents and the time and the sequence of administration seem to be the key factors for a successful treatment. In the cases reported here, we intended to target different points of the same cellular pathway or different pathways in order to offer the patients the maximum therapeutic advantage, given the lack of comprehensive guidelines at the time of treatment. We tend to attribute the long-term survival achieved to the sequential medical treatment. Recent studies suggest that, even after a VEGFR inhibitor failure, a switch to another VEGFR inhibitor could still be effective given that the targets are overlapping but not identical [28]. Additionally, a failure of a previous anti-VEGF therapy might not preclude failure of a VEGFR inhibition given the activity seen using sunitinib in patients refractory to bevacizumab. This theory could be consistent with our experience.

Resistance to anti-VEGF therapy may arise through the development of alternative angiogenic pathways. A proposed strategy to overcome resistance is to combine antiangiogenic agents with different mechanisms of action [19]. Thus, case 1 was treated with a sorafenib and bevacizumab doublet to overcome the eventual resistance caused by previous treatment with sunitinib.

Also, the interdisciplinary task force of the German Cancer Society (DKG) suggested that targeted therapies should strive for a sufficient treatment duration in each line of therapy in order to achieve the best therapeutic outcome [29], whereas Staehler *et al*. [2] suggested that systemic antiangiogenic therapy should be continued even if there is no evidence of disease.

Kirchner *et al*. [27] proposed a patient-based treatment strategy based mainly on the tumor burden and the development of the disease to try to find a balance between tumor burden and quality of life and thus to obtain the best outcome for the patient. In this study, patients with a high tumor burden were treated with sunitinib, whereas sorafenib was preferred when tumor control was the main focus, conferring better quality of life.

According to the pace of the disease, the cases in our study were under careful surveillance and were offered best supportive care after being treated with metastasectomies, since the residual tumor burden was not life-threatening. Both cases were treated with sunitinib at their further relapse targeting a high remission rate.

From our experience, we could possibly attribute the long-term survival of the patients in part to the multimodal treatment and in part to the use of multiple therapeutic agents. The clinical strategy to treat RCC patients who have a solitary metastasis and a good performance status with metastasectomy and continuous sequential therapy with cytokines and new targeted agents could be given consideration according to the patient’s individual characteristics and current guidelines.

Beyond overall survival and PFS, it is fundamental to distinguish which therapies offer a major benefit in terms of quality of life. Only a few studies have reported an advantage in quality of life when VEGFR inhibitors are administered. Cella *et al*. [30] and Escudier *et al*. [31] reported improved quality of life in patients treated with sunitinib and sorafenib, respectively. In case 1, the dramatic improvement in quality of life could be attributed to the multimodal character of treatment, which consisted of an orthopedic surgical procedure followed by radiotherapy and systemic cytokine-based chemotherapy. mRCC should be broadly considered a systematic disease demanding multimodal treatment that should ideally be coordinated by a multidisciplinary working group in experienced centers.

## Conclusions

Patients with untreated mRCC have a 5-year survival rate of less than 20%. Several treatment modalities that may prolong survival and improve quality of life are at our disposal. Here, we present two patients whose RCC was treated with nephrectomy, metastasectomies, and sequential systemic therapy and who experienced a very satisfactory quality of life and long-term survival. Metastasectomy, when feasible, could be considered a clinical option, especially in patients with favorable characteristics. Sequential medical therapy based on cytokines and on an array of targeted treatment agents such as tyrosine kinase receptor inhibitors, humanized monoclonal antibodies against vascular endothelial growth factor, and mTOR inhibitors may be established as a routine practice in the treatment of mRCC in selected patients. The effects of multimodal treatment and sequential use of established or experimental targeted agents should be studied further. Ideally, randomized clinical trials and open-label trials that reflect real-life clinical practice with endpoints that include quality of life and prevention of disability should be undertaken in order to optimize our current treatment strategy. It is conceivable that current guidelines could be amended, leading to an increase in the number of RCC patients who achieve long-term survival and an acceptable quality of life.

## Abbreviations

CT, computed tomography; IFN-α, interferon-alpha; mRCC, metastatic renal cell carcinoma; mTOR, mammalian target of rapamycin; PDGFR, platelet-derived growth factor receptor; PFS, progression-free survival; RCC, renal cell carcinoma; RTK, receptor tyrosine kinase; VEGF, vascular endothelial growth factor; VEGFR, vascular endothelial growth factor receptor.

## Competing interests

The authors declare that they have no competing interests.

## Authors’ contributions

JS and GK participated in collection and interpretation of clinical and laboratory data and drafted the manuscript. NT coordinated and helped to draft the manuscript. All authors read and approved the final manuscript.

## Consent

Written informed consent was obtained from the wife of the first patient and directly from the second patient for publication of this case report and accompanying images. A copy of the written consent is available for review by the Editor-in-Chief of this journal.

## References

[B1] Herrmann E Bierer S Wulfing C Update on systemic therapies of metastatic renal cell carcinoma World J Urol 2010 28 303 309 10.1007/s00345-010-0519-5 20180125

[B2] Staehler M Haseke N Zilinberg E Stadler T Karl A Siebels M Dürr HR Siegert S Jauch KW Bruns CJ Stief CG Complete remission achieved with angiogenic therapy in metastatic renal cell carcinoma including surgical intervention Urol Oncol 2010 28 139 144 10.1016/j.urolonc.2009.03.033 19576802

[B3] Chen F Fujinaga T Shoji T Miyahara R Bando T Okubo K Hirata T Date H Pulmonary resection for metastasis from renal cell carcinoma Interact Cardiovasc Thorac Surg 2008 7 825 828 10.1510/icvts.2008.181065 18593745

[B4] Athar U Gentile TC Treatment options for metastatic renal cell carcinoma: a review Can J Urol 2008 15 3954 3966 18405442

[B5] Bellmunt J Future developments in renal cell carcinoma Ann Oncol 2009 20 Suppl 1 i13 i17 19430003 10.1093/annonc/mdp074

[B6] Escudier B Goupil MG Massard C Fizazi K Sequential therapy in renal cell carcinoma Cancer 2009 115 10 Suppl 2321 2326 19402067 10.1002/cncr.24241

[B7] Cheng L Zhang S MacLennan GT Lopez-Beltran A Montironi R Molecular and cytogenetic insights into the pathogenesis, classification, differential diagnosis, and prognosis of renal epithelial neoplasms Hum Pathol 2009 40 10 29 10.1016/j.humpath.2008.09.009 19027455

[B8] Sene AP Hunt L McMahon RF Carroll RN Renal carcinoma in patients undergoing nephrectomy: analysis of survival and prognostic factors Br J Urol 1992 70 125 134 10.1111/j.1464-410X.1992.tb15689.x 1393433

[B9] Flanigan RC Salmon SE Blumenstein BA Bearman SI Roy V McGrath PC Caton JR Jr Munshi N Crawford ED Nephrectomy followed by interferon alfa-2b compared with interferon alfa-2b alone for metastatic renal-cell cancer N Engl J Med 2001 345 1655 1659 10.1056/NEJMoa003013 11759643

[B10] Ruiz-Cerda JL Jimenez Cruz F Surgical treatment for renal cancer metastases Actas Urol Esp 2009 33 593 602 19658314 10.1016/s0210-4806(09)74194-4

[B11] Lin PP Mirza AN Lewis VO Cannon CP Tu SM Tannir NM Yasko AW Patient survival after surgery for osseous metastases from renal cell carcinoma J Bone Joint Surg Am 2007 89 1794 1801 10.2106/JBJS.F.00603 17671020

[B12] Althausen P Althausen A Jennings LC Mankin HJ Prognostic factors and surgical treatment of osseous metastases secondary to renal cell carcinoma Cancer 1997 80 1103 1109 10.1002/(SICI)1097-0142(19970915)80:6<1103::AID-CNCR13>3.0.CO;2-C 9305711

[B13] Kavolius JP Mastorakos DP Pavlovich C Russo P Burt ME Brady MS Resection of metastatic renal cell carcinoma J Clin Oncol 1998 16 2261 2266 9626229 10.1200/JCO.1998.16.6.2261

[B14] Volkmer BG Gschwend JE Value of metastases surgery in metastatic renal cell carcinoma Urologe A 2002 41 225 230 10.1007/s00120-002-0204-4 12132271

[B15] Pomer S Klopp M Steiner HH Brkovic D Staehler G Cabillin-Engenhart R Brain metastases in renal cell carcinoma. Results of treatment and prognosis Urologe A 1997 36 117 125 10.1007/s001200050076 9199038

[B16] Coppin C Porzsolt F Awa A Kumpf J Coldman A Wilt T Immunotherapy for advanced renal cell cancer Cochrane Database Syst Rev 2005 1 CD001425 15674877 10.1002/14651858.CD001425.pub2

[B17] Guida M Colucci G Immunotherapy for metastatic renal cell carcinoma: is it a therapeutic option yet? Ann Oncol 2007 18 Suppl 6 vi149 vi152 17591810 10.1093/annonc/mdm245

[B18] Schmidinger M Steger GG Budinsky AC Wenzel C Brodowicz T Locker GJ Kramer G Marberger M Zielinski CC Vinorelbine and interferon-alpha2c as second-line therapy in metastatic renal cell carcinoma Anticancer Drugs 2000 11 175 179 10.1097/00001813-200003000-00005 10831276

[B19] Moreno Garcia V Basu B Molife LR Kaye SB Combining antiangiogenics to overcome resistance: rationale and clinical experience Clin Cancer Res 2012 18 3750 3761 10.1158/1078-0432.CCR-11-1275 22547772

[B20] Godley PA Stinchcombe TE Renal cell carcinoma Curr Opin Oncol 1999 11 213 217 10.1097/00001622-199905000-00014 10328597

[B21] Rini BI Halabi S Rosenberg JE Stadler WM Vaena DA Ou SS Archer L Atkins JN Picus J Czaykowski P Dutcher J Small EJ Bevacizumab plus interferon alfa compared with interferon alfa monotherapy in patients with metastatic renal cell carcinoma: CALGB 90206 J Clin Oncol 2008 26 5422 5428 10.1200/JCO.2008.16.9847 18936475 PMC2651074

[B22] Motzer RJ Hutson TE Tomczak P Michaelson MD Bukowski RM Rixe O Oudard S Negrier S Szczylik C Kim ST Chen I Bycott PW Baum CM Figlin RA Sunitinib versus interferon alfa in metastatic renal-cell carcinoma N Engl J Med 2007 356 115 124 10.1056/NEJMoa065044 17215529

[B23] Escudier B Eisen T Stadler WM Szczylik C Oudard S Siebels M Negrier S Chevreau C Solska E Desai AA Rolland F Demkow T Hutson TE Gore M Freeman S Schwartz B Shan M Simantov R Bukowski RM TARGET Study Group Sorafenib in advanced clear-cell renal-cell carcinoma N Engl J Med 2007 356 125 134 10.1056/NEJMoa060655 17215530

[B24] Sternberg CN Davis ID Mardiak J Szczylik C Lee E Wagstaff J Barrios CH Salman P Gladkov OA Kavina A Zarbá JJ Chen M McCann L Pandite L Roychowdhury DF Hawkins RE Pazopanib in locally advanced or metastatic renal cell carcinoma: results of a randomized phase III trial J Clin Oncol 2010 28 1061 1068 10.1200/JCO.2009.23.9764 20100962

[B25] Hudes G Carducci M Tomczak P Dutcher J Figlin R Kapoor A Staroslawska E Sosman J McDermott D Bodrogi I Kovacevic Z Lesovoy V Schmidt-Wolf IG Barbarash O Gokmen E O'Toole T Lustgarten S Moore L Motzer RJ Global ARCC Trial Temsirolimus, interferon alfa, or both for advanced renal-cell carcinoma N Engl J Med 2007 356 2271 2281 10.1056/NEJMoa066838 17538086

[B26] Motzer RJ Escudier B Oudard S Hutson TE Porta C Bracarda S Grünwald V Thompson JA Figlin RA Hollaender N Urbanowitz G Berg WJ Kay A Lebwohl D Ravaud A RECORD-1 Study Group Efficacy of everolimus in advanced renal cell carcinoma: a double-blind, randomised, placebo-controlled phase III trial Lancet 2008 372 449 456 10.1016/S0140-6736(08)61039-9 18653228

[B27] Kirchner H Strumberg D Bahl A Overkamp F Patient-based strategy for systemic treatment of metastatic renal cell carcinoma Expert Rev Anticancer Ther 2010 10 585 596 10.1586/era.10.25 20397923

[B28] Stein MN Flaherty KT CCR drug updates: sorafenib and sunitinib in renal cell carcinoma Clin Cancer Res 2007 13 3765 3770 10.1158/1078-0432.CCR-06-2844 17606705

[B29] Jager E Monitoring of metastatic renal cell carcinoma - standards and challenges Onkologie 2010 33 Suppl 1 15 17 Article in German 20164672 10.1159/000265685

[B30] Cella D Li JZ Cappelleri JC Bushmakin A Charbonneau C Kim ST Chen I Motzer RJ Quality of life in patients with metastatic renal cell carcinoma treated with sunitinib or interferon alfa: results from a phase III randomized trial J Clin Oncol 2008 26 3763 3769 10.1200/JCO.2007.13.5145 18669464

[B31] Escudier B Szczylik C Hutson TE Demkow T Staehler M Rolland F Negrier S Laferriere N Scheuring UJ Cella D Shah S Bukowski RM Randomized phase II trial of first-line treatment with sorafenib versus interferon Alfa-2a in patients with metastatic renal cell carcinoma J Clin Oncol 2009 27 1280 1289 10.1200/JCO.2008.19.3342 19171708

